# Love as Bait: A Scoping Review and Crime Script Analysis of Online Romance Scams

**DOI:** 10.1177/15248380251361046

**Published:** 2025-09-09

**Authors:** Janneke M. Schokkenbroek, Thom Snaphaan

**Affiliations:** 1 Delft University of Technology, Delft, The Netherlands; 2 Inholland University of Applied Sciences, The Hague, The Netherlands; 3 Ghent University, Ghent, Belgium; 4 Avans University of Applied Sciences, ‘s-Hertogenbosch, The Netherlands

**Keywords:** online romance scams, online dating fraud, crime script analysis, scoping review, modus operandi

## Abstract

This study presents a scoping review and crime script analysis of the modus operandi of online romance scammers. Online romance scams are a form of fraud in which perpetrators fabricate online romantic relationships with victims, aiming to emotionally manipulate and, ultimately, financially exploit them. The review aims to synthesize existing research on how scammers operate and to develop a comprehensive crime script that can guide prevention and policy efforts. A literature search was conducted in Web of Science and Scopus. The search yielded 318 initial results, which were screened for relevance using ASReview Lab and supplemented with 14 additional sources from reference lists and Google Scholar. In total, 50 empirical studies were included based on their descriptions of scammer behaviors. Data were analyzed by coding relevant passages on scammer actions and process models, which were then categorized into scenes and actions to construct a crime script. The resulting script identifies nine major scenes in the scam process: (1) preparation (the Setup); (2) target selection (the Hunt); (3) initial contact (the Hook);(4) transition to private communication (the Shift); (5) grooming; (6) the Sting; (7) financial transaction (the Payout); (8) the Squeeze (e.g., sextortion); and (9) the Aftermath (e.g., revictimization). Each scene includes multiple possible actions and variations, demonstrating the flexibility and adaptability of scammers. The review underscores gaps in previous process models by highlighting non-linearity, scammer adaptability, and revictimization in the online romance scam process. This study contributes to both theory and practice by offering a detailed framework for understanding and reducing (the harm following) online romance scams.

## Introduction

=======================================================

***Ways of saying hi to a client Pick one below***

=======================================================

***Hi, I hope you’re having a great day.***

***Saw your profile and just had to say hi.***

***Hey, what are you up to right now?***

***[. . .]***

*Excerpt from a ‘playbook’ of online romance scammers* (SocialCatfish, 2023).

With the rise of online dating platforms and social media, it has become easier than ever to find potential romantic partners, even across geographic borders (Finkel et al., 2012). While these platforms can facilitate genuine connections, they also provide a breeding ground for criminal activity, most notably *online romance scams*. This type of fraud is a complex and damaging problem, where scammers pretend to want to establish a genuine romantic relationship with their victim, while the real goal is to financially exploit them (Cross et al., 2018).

The consequences of online romance scams are far-reaching, both financially and emotionally. A recent cybercrime report from the Federal Trade Commission (2021) revealed that financial losses resulting from online romance fraud have accumulated to approximately $956 million, ranking it third among cybercrimes and surpassing all other fraud categories in terms of monetary impact. In 2024, nearly 59,000 Americans lost an estimated $697.3 million to romance scammers. In our home countries, Belgium and the Netherlands, online romance scams are also on the rise. In 2023, as many as 494 cases of online romance scams were registered in the Netherlands. Of these, 264 victims also suffered financial damage, with a total registered loss of €7,644,870 (Fraudehelpdesk, 2024). This amounts to an average loss of nearly €29,000 per victim, an amount that leads to long-term financial problems for many. Although comparable figures for Belgium are lacking, there are reports of victims who lost significant amounts, in exceptional cases as high as €120,000 (Cauwenberghs & Van Bakel, 2023).

In addition to these financial losses, victims also experience serious emotional consequences. Common consequences include sadness, loneliness, anger, shame, distrust, a damaged self-image, and long-term social and psychological problems (Buchanan & Whitty, 2014; Niman et al., 2023; Whitty & Buchanan, 2016). Some victims also report physical symptoms, such as sleep deprivation, hair loss, and weight gain (Cole, 2024). In extreme cases, victims even report post-traumatic stress and suicidal thoughts (Cole, 2024; Whitty & Buchanan, 2016). The emotional impact is often intensified by a lack of social support. Victims may feel ashamed or afraid to share their experiences with others, and when they do, they are often met with misunderstanding, anger, or reproach (Cross, 2015; Whitty & Buchanan, 2016). This phenomenon of *victim blaming*—not only placing responsibility on the victim for what happened but also questioning their judgment—deepens the harmful consequences of the scam and raises the threshold for victims to report their experiences to the police or other authorities (Meikle & Cross, 2024).

Moreover, victims must also grapple with the emotional devastation of losing what they believed was a genuine romantic relationship. In fact, many victims find this the most difficult loss to deal with, more so than the financial loss (Whitty, 2015). This “double hit” (Whitty & Buchanan, 2012) of both financial and emotional loss makes this form of fraud uniquely destructive.

### What Is an Online Romance Scam?

Online romance scams often follow a similar pattern (Choi et al., 2024; Shaari et al., 2019; Whitty, 2015). The fraud usually begins with the creation of credible fake identities on social media or online dating platforms, with scammers using stolen or fabricated photos and creating carefully constructed profiles. They then develop deep emotional bonds with their victims through intense communication, aimed at building trust and emotional dependence. This is eventually followed by financial solicitations, which often escalate in both frequency and extent, sometimes costing victims significant sums of money.

While significant progress has been made in understanding the broader mechanisms of this form of fraud, crucial gaps remain. Notably, an integrated and detailed overview of the modus operandi of online romance scammers is currently lacking. Such an overview is essential to gain a more complete picture of how this fraud develops, what patterns are consistent, and where variations occur. These insights are critical to developing effective prevention strategies and to better support victims. Moreover, such an overview contributes to the broader scientific discussion of digital forms of fraud and the social dynamics that enable them.

### Crime Scripting

Crime script analysis, or crime scripting, is a promising method for understanding complex crimes (Cornish, 1994; Dehghanniri & Borrion, 2021). It offers a structured framework to map out the criminal process in sequence, from preparation to completion and the aftermath, providing a step-by-step understanding of how a specific form of crime is carried out. A crime scriptnot only records the actions taken by offenders but also the tools and strategies they use in the process (Snaphaan, 2025). Each “scene” describes a phase (with a subgoal) of the criminal process. Within a scene, offenders may achieve their goals in various ways. These alternative methods are called *equifinal actions* (Abelson, 1981), meaning they lead to the same outcome but take different routes to get there.

Crime scripts can exist at different levels of abstraction. *Metascripts* refer to broad categories of crime (e.g., fraud in general), *protoscripts* describe a more concrete form of the metascript (e.g., dating fraud), and the *script level* contains a detailed breakdown of the sequential steps of the criminal process (e.g., online romance scams), including variations (equifinal actions) within each scene. At the most specific level, *tracks* illustrate how a specific modus operandi plays out by selecting and combining the variations identified at the script level. For instance, tracks show how a scam may adapt to a particular context, such as the platform used or the type of victim targeted (Cornish, 1994). Scripts can also have horizontal and vertical relationships: horizontally, they link related crimes (e.g., romance scams and identity theft); vertically, they connect abstract strategies to concrete actions, referring to the different abstraction levels of crime scripts (Gómez-Quintero et al., 2023; Snaphaan, 2025).

Generating and organizing knowledge about the modus operandi of criminals is essential in gaining deeper understanding of offender behavior and the procedural aspects of crime. Mapping out how offenders carry out specific forms of crime provides insights that can inform strategies for interventions and specific (situational) crime prevention measures. Crime scripting has been successfully applied in previous research to analyze a wide range of criminal activities (Dehghanniri & Borrion, 2021; Snaphaan & Klerks, 2024) and has proven valuable in the broader context of online fraud (Leclerc & Morgenthaler, 2023; Loggen & Leukfeldt, 2022; Matthijsse et al., 2023). Notably, Dehghanniri and Borrion’s (2021) systematic review identified that the majority of existing crime scripts focus on cybercrime (for more recent examples, see also Leclerc et al., 2021; O’Malley et al., 2023)—a trend that is unsurprising given the relatively recent emergence of these forms of offending, and the resulting need for a deeper understanding of how such crimes unfold. Several studies have used crime script analysis to map specific types of online scams, such as customer-to-customer e-commerce fraud (Lee, 2022), bank card fraud and phone scams (Van Nguyen, 2022), and computer fraud (Lwin Tun & Birks, 2023).

### Purpose of This Study

The goal of this study is to compile and structure key findings on the modus operandi of online romance scammers. To achieve this, we conduct a scoping review of the existing literature. First, we present an overview of existing process models of online romance scams, which are then evaluated for similarities, inconsistencies, and limitations. Second, using this analysis, supplemented by detailed information on how scammers operate from prior empirical studies, we develop a comprehensive crime script. This script provides a detailed overview of the various steps and strategies within this form of fraud. It not only provides a valuable basis for future research but also offers concrete guidance for policy and practice by identifying intervention options that can protect victims from this deeply impactful crime.

Beyond its relevance for crime prevention, policy, and research, this study also contributes to a deeper understanding of the emotional and psychological harm caused by online romance scams. By mapping out the stages and tactics used in these scams, this review provides critical insights for mental health professionals, victim support organizations, and trauma-informed care providers. The findings of this scoping review and crime script analysis can help identify patterns of abuse, validate victims’ experiences, and inform tailored therapeutic and preventative interventions aimed at mitigating the long-term impact of this not only financially but—perhaps even more so—emotionally exploitative form of cybercrime.

## Method

### Scoping Review

A scoping review was conducted to create a comprehensive overview of the modus operandi of online romance scammers. Unlike systematic reviews, which are typically guided by narrowly defined research questions and apply strict criteria for inclusion, quality appraisal, and synthesis, scoping reviews are broader in scope and aim to map key concepts, types of evidence, and knowledge gaps (Munn et al., 2018). They are designed to map the landscape of available evidence without necessarily assessing the quality of individual studies or synthesizing results through a more statistical approach.

In our study, a scoping review was the most appropriate approach because the literature on online romance scams spans diverse methods and disciplinary perspectives and includes a wide variety of descriptions of scammer strategies and process models. Our objective was to gather, organize, and synthesize these varied insights to generate a comprehensive and integrated crime script, not to test a predefined hypothesis.

### Data Collection and Selection Process

**Figure 1.**
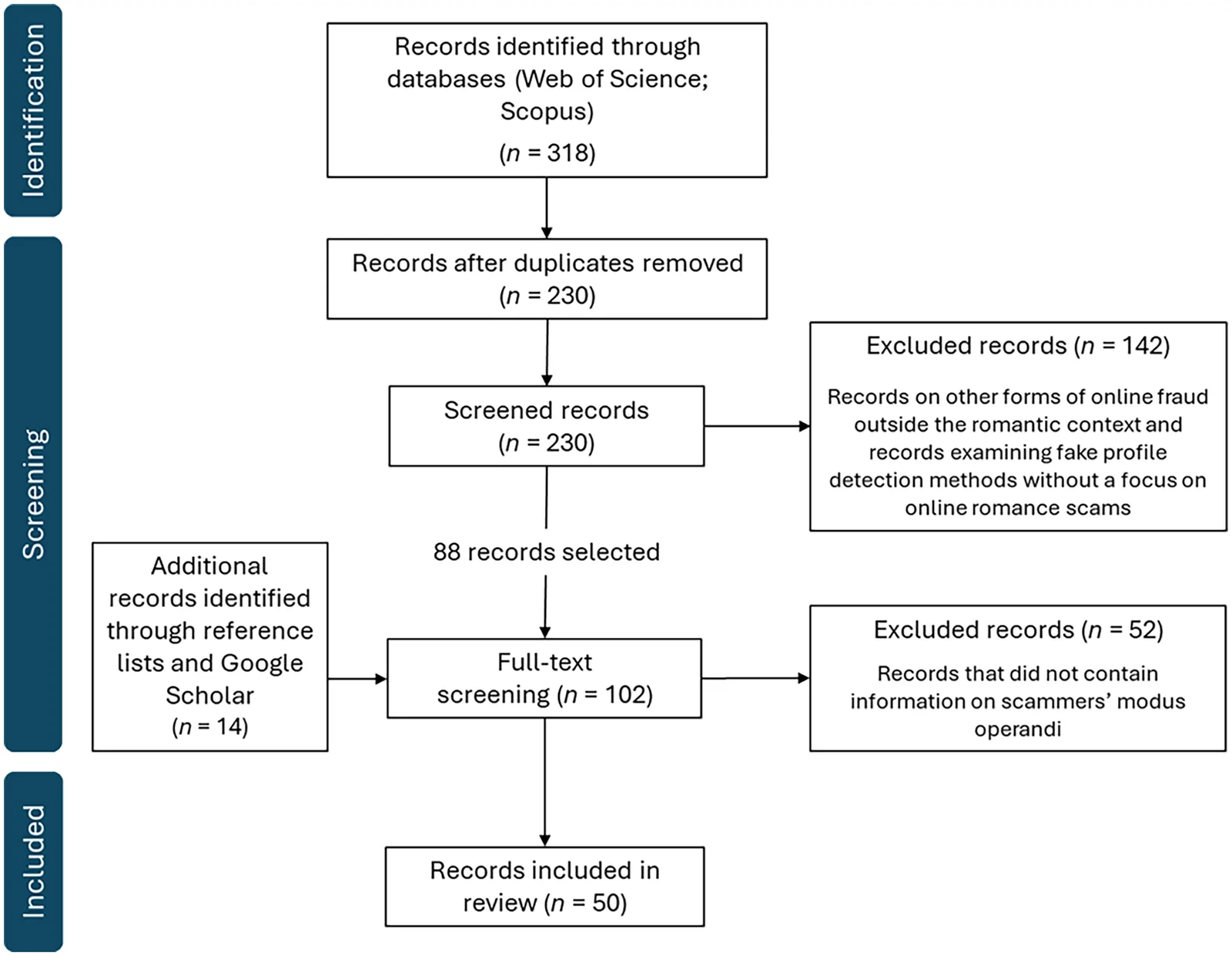
PRISMA flow diagram of the literature selection process.

The literature search was conducted in two scholarly databases: Web of Science and Scopus. The search terms were based on the three-key elements of the term “online romance scam,” as shown in Table 1. Figure 1 presents a PRISMA flow diagram of the literature selection process.

**Table 1.** Overview of Search Terms and Boolean Operators Used in the Scoping Review.

Topic	Search Terms & Boolean Operators
online	(online OR internet OR cyber OR electronic)
	AND
romance	(romance OR dating OR romantic OR love)
	AND
scam	(scam OR fraud OR swindle OR catfish)

The search was conducted on November 13, 2024 and returned a total of 318 articles: 168 from Scopus and 150 from Web of Science. After removing duplicates, 230 unique records remained. The first round of screening focused on the titles and abstracts of these 230 records and was conducted using ASReview Lab, a tool that accelerates manual screening with machine learning support (Van de Schoot et al., 2021). This phase excluded records that were not directly relevant to the study, such as studies on other forms of online fraud outside the romantic context, such as *phishing* or *mobile gambling scams*. Studies on detection methods for fake profiles that did not specifically focus on online romance scammers were also excluded. After this screening, 88 records remained for full-text screening. In addition, 14 more relevant records were identified through reference lists and an additional search on Google Scholar (conducted on January 5, 2025). This increased the total number of records for full-text review to 102.

During the full-text screening, records were evaluated for their relevance, with an emphasis on studies that provide insight into the modus operandi of online romance scammers. For example, articles that focused exclusively on the perspective of victims or on prevention strategies without detailed information on the modus operandi of scammers were excluded. Finally, 50 records were selected that contained relevant and detailed information about the behavior of online romance scammers. A summary of the included records is provided in Appendix I.

### Data Analysis

The 50 selected records were imported into Zotero (version 7.0.11; Corporation for Digital Scholarship, 2025), where they were systematically annotated. This was done in a step-wise manner. First, we annotated and analyzed records (*n* = 10) that outlined existing models of online romance scams. Passages outlining existing models of online romance scams were analyzed descriptively rather than coded, as our objective was to compare the models as holistic representations of scam processes.

Second, we annotated and analyzed passages that provided insight into the modus operandi of scammers across all records (*n* = 50). These passages that provided insights into the specific strategies and actions of scammers were subjected to a structured coding process, as these findings served as the empirical basis for constructing the crime script. Specifically, the coding process combined deductive and inductive elements. We began with a preliminary framework based on the ten existing process models we identified, which provided an outline of potential scenes and actions. However, this structure was refined and expanded through iterative, inductive coding of the full texts. Relevant passages were coded and categorized into the scenes initially identified, as well as used to identify additional scenes and actions that were not captured in the preliminary framework. This allowed us to both consolidate recurring elements across models and incorporate additional strategies or variations that had not been captured in prior frameworks. Through this process, nine scenes were identified. These categories formed the structure of the final crime script.

## Results

### Existing Models of Online Romance Scams

Ten prior studies have introduced models to describe the process of online romance scams. Despite variations among these models, they generally share a similar structure, with phases^1^ of approach, manipulation, and exploitation. Appendix II provides an overview of these existing process models, including the scenes and elements distinguished within them. In what follows, we discuss common scenes across these models, as well as what inconsistencies and limitations can be noted upon comparing these process models.

#### Common Scenes

While not all process models of online romance scams are structured into scenes or use uniform terminology, certain key scenes recur across most models. At the same time, it is important to emphasize that some scenes are missing from certain models or are subject to debate due to inconsistent findings. For example, some researchers found no evidence of sextortion in the context of online romance scams and therefore did not include this scene in their process model (Smeitink, 2021), while others have shown that these practices do occur and gave this scene a central role in their model (Whitty, 2019).

The scenes described below represent the steps that are common across most process models and therefore form a shared foundation. This does not mean, however, that the overview is exhaustive or includes all possible scenes within an online romance scam. Additional scenes are listed in Appendix II, and our crime script analysis will explore and contextualize them further.

##### Common Scene 1: Initiation

In most models, an online romance scam begins with the initiation of contact (e.g., Anesa, 2020; Dickerson et al., 2020). This typically takes place through dating platforms, social media, or email. Scammers create carefully crafted personas and online profiles designed to appear credible and appealing to potential victims (e.g., Shaari et al., 2019; Whitty, 2013).

##### Common Scene 2: Building Trust (the “Grooming” Scene)

During this scene, scammers engage in intensive communication with victims to build emotional attachment and trust (Shaari et al., 2019). This can be a prolonged process, sometimes lasting months or even years. They use various grooming techniques such as frequent compliments, emphasizing shared interests, expressing romantic feelings, and even sending gifts (e.g., Huhn, 2023).

##### Common Scene 3: Financial Exploitation

Once trust has been established, scammers introduce a narrative aimed at obtaining financial support from the victim. This often begins with a small request (e.g., a contribution for a plane ticket) to lower the barrier for larger transfers, an approach known as the “foot-in-the-door” technique (Whitty, 2013). In most cases, scammers fabricate a crisis situation, such as a medical emergency, legal trouble, or travel restrictions, to create a sense of urgency and persuade victims to provide financial assistance (Anesa, 2020; Whitty, 2013).

#### Inconsistencies and Limitations

Although prior studies offer valuable insights into online romance scams, existing models show notable inconsistencies and limitations, which hinder a comprehensive understanding of this type of fraud. Key issues relate to (a) the number and interpretation of scenes, (b) the focus of process models, and (c) the level of detail.

##### (a) Number and Interpretation of Scenes

Models vary significantly in the number and content of scenes. Some outline only a few steps (e.g., Wang & Zhou, 2023), while others define up to 16 (e.g., Shaari et al., 2019). Moreover, critical steps—such as gathering victim information, repeated victimization, or the scam’s conclusion—are often omitted. Links to related crimes like identity theft, money laundering, and sextortion are also frequently overlooked, despite evidence from prior research (Cross et al., 2023; Cross & Holt, 2023; Huhn, 2023; Ray & Henry, 2025; Rege, 2009).

In addition, most models assume a linear sequence (e.g., Andoh-Baidoo et al., 2024; Choi et al., 2024; Whitty, 2013, 2019), assuming a fixed order of events. However, others propose a non-linear approach, where scammers revisit or adapt scenes based on victim responses (Anesa, 2020; Huhn, 2023). Empirical studies support this flexible model, showing that scammers often cycle through steps to maintain control (Huhn, 2023; Koon & Yoong, 2017).

##### (b) Focus Regarding Perspective, Operational Structure, and Variants of Online Romance Scams

Another limitation is the varying focus of studies. Some emphasize the victim’s perspective (Whitty, 2015, 2019), others the offender’s strategies (Anesa, 2020; Shaari et al., 2019), and a few integrate both (Huhn, 2023). These differing angles in existing models limit the ability to develop a comprehensive understanding of the process.

Operational structures are also treated differently: some studies consider individual offenders (Whitty, 2013, 2015), while others focus on organized networks (Andoh-Baidoo et al., 2024; Wang & Zhou, 2023). However, scammers may work alone, in small teams, or as part of large networks (Rege, 2013), requiring broader analysis.

Lastly, models also vary in their focus on specific variants of online romance scams. For example, some examine the *Sha Zhu Pan* scam (“pig-butchering”), which is particularly prevalent in China and Southeast Asia (Wang & Zhou, 2023), while others take a broader approach or zoom in on specific cultural contexts, such as South Korea (Choi et al., 2024). While these approaches are valuable, they contribute to a fragmented overview, as there is currently no integrated model that captures both shared and context-specific elements of online romance scams.

##### (c) Level of Detail3

A final limitation is the variable level of detail in existing models. Many models remain superficial in their descriptions of scammers’ decisions and modus operandi. For example, while many process models describe the grooming phase, they do not address the specific choices and variations within this step (Anesa, 2020; Whitty, 2013, 2015, 2019). Conversely, other studies focus in great detail on specific persuasion techniques within specific scenes (Wang & Zhou, 2023), while other strategies or steps in the process are then understudied. Another example is that only some models address the technologies used by scammers (Andoh-Baidoo et al., 2024), while other models ignore these altogether. These inconsistencies make it difficult to grasp the full complexity of online romance scams.

The described inconsistencies and limitations may have various causes, but methodological differences are a key factor. The ten existing process models use varied methods, such as in-depth interviews with victims or law enforcement agencies, or analyses of police reports, text message exchanges between offenders and victims, and victim testimonies on online platforms. None included input directly from offenders. Moreover, not all cases are reported to the police. Whether victims choose to report an incident is influenced by a range of factors, meaning that only a portion of incidents are officially recorded. These methodological differences and blind spots raise concerns about the representativeness of available data and underscores the need for a more integrated, multifaceted research approach.

### Crime Script of the Modus Operandi of Online Romance Scammers

**Figure 2.**
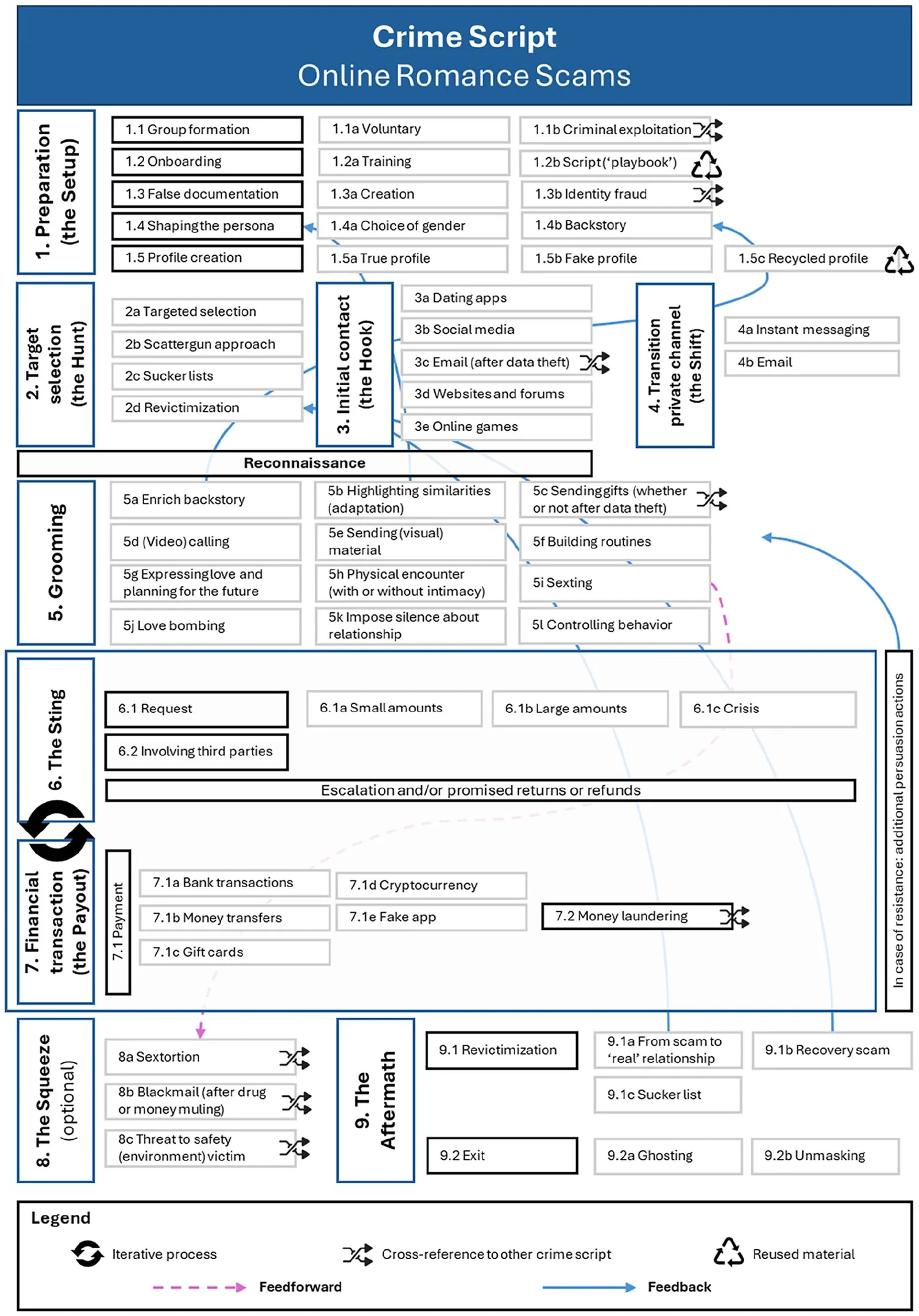
Crime script of online romance scams based on a scoping review of 50 studies.

To address the gaps and inconsistencies in previous models, a detailed and comprehensive overview was needed—one that combines the strengths and shared elements of existing models while also accounting for variations in scenes and specific actions. For this reason, we conducted an extensive crime script analysis based on the findings from prior research on online romance scams. In addition to analyzing the ten existing process models, we also included the remaining 40 studies in our crime script analysis. These empirical studies each provide insight into the modus operandi of scammers. The analysis of all 50 studies resulted in a crime script that systematically maps out the various ways in which online romance scammers operate. A schematic representation of this script can be found in Figure 2.

#### Descriptive Insights From Scoping Review

Before presenting the crime script analysis, we share several descriptive insightsfrom our scoping review to provide context on scam duration and scammer characteristics. Online romance scams are often long-term, with scammers building trust over months or even years. While some scams last only a few days (Gillespie, 2017; Wang & Zhou, 2023), most span several months (Rege, 2009; Whitty, 2013), and some continue for years (Huhn, 2023)—in extreme cases, up to 8 years (Aborisade et al., 2024).

Furthermore, scammers can be male or female (Khader & Yun, 2017) and are often located in West Africa, particularly Nigeria, Ghana, and Malaysia. About 50% are based in West Africa (Edwards et al., 2018). Despite their actual locations, scammers typically present themselves as white individuals from the United States (Edwards et al., 2018).

#### Crime Script Analysis

In our crime script analysis, we identified nine scenes that make up the modus operandi of online romance scammers, each containing multiple actions and variations. For example, in Scene 1 (Preparation), Action 1.3 involves creating false documentation. This can be in the form of fake passports or bank statements (1.3a), or by committing identity fraud using someone else’s personal documents (1.3b). These actions can occur individually, together, or not at all. A scammer might use only fake documents, only identity fraud, both, or neither—when no fake documentation is used at all. This flexibility applies across all actions: some scammers skip certain steps, like joining a group or undergoing a training period, while others carry out multiple actions. However, some actions, such as creating an online profile and persona, are almost universal.

This highlights that the crime script represents a range of possible actions and variations, not a fixed sequence or checklist. The following section provides a detailed explanation of each scene and action.^2^

#### Scene 1. Preparation (the Setup)

Online romance scammers thoroughly prepare their scams. The first step in this preparation is *group formation (1.1)*. While some scammers operate independently, many work within organized groups (e.g., Rege, 2013). Numerous scam organizations exist globally, such as the *Sha Zhu Pan* scam in China and Southeast Asia, which is exclusively carried out in an organized setting (Wang & Zhou, 2023). In most cases, scammers *voluntarily* join such groups (*1.1a*) after being recruited by other scammers. However, there are also cases—particularly within the *Sha Zhu Pan* scam—where scammers were initially victims themselves, lured into criminal organizations under false pretenses, and later *forced* to work as scammers (*1.1b*; Wang, 2024).

Within romance scam organizations, scammers are *inducted* in various ways (*1.2*). During formal *training* sessions (*1.2a*), they learn essential skills such as identifying suitable targets, initiating conversations, using digital tools, and crafting effective financial requests (Barnor, 2024; Barnor et al., 2020; Cassiman, 2019; Wang & Topalli, 2024a). Some organizations also provide detailed *scripts or “playbooks”*^3^ (*1.2b*), which include specific scenarios and instructions on what the scammer should say or how to respond (Choi et al., 2024; Huhn, 2023; Yen & Jakobsson, 2016).

Another preparatory action is the previously discussed creation of *false documentation* (*1.3*). Scammers *fabricate documents* (*1.3a*) such as passports, certificates, or bank statements to enhance their credibility (Smeitink, 2021). In some cases, this involves *identity fraud* (*1.3b*), where the identity or personal data of others—often previous victims—are misused (Huhn, 2023).

The next action is the *creation of the persona* (*1.4*). Scammers decide which identity to assume, including *gender* (*1.4a*) and *backstory* (*1.4b*). These choices are strategic, as they influence the type of target they attract and determine the narrative used later to extract money (Anesa, 2020; Whitty, 2013). The created persona is not only strategic, but also flexible—scammers frequently adapt their profile and backstory based on the personal information they gather about (potential) targets, whether through public profile data or during initial interactions. This adaptive process is visualized in the crime script with a feedback loop arrow.

The final action in the preparation is the *creation of a profile* based on the persona (*1.5*). In rare cases, a scammer uses their *own image* (*1.5a*; Gillespie, 2017; Wang & Lu, 2007), but typically, a *fake profile* (*1.5b*) is created (e.g., Rege, 2013). To do this, scammers often use photos of real individuals, such as previous victims (Huhn, 2023), random social media users (Rege, 2013; Whitty, 2015), or models (Rege, 2013). Increasingly, AI-generated images—commonly known as *deepfakes*—are being used (Cross, 2022; Dickerson et al., 2020; Wiederhold, 2024). Scam profiles are frequently *recycled* (*1.5c*), where previously used profiles are reused or appear multiple times on the same platform with only minor edits to profile details (Huang et al., 2015; Pizzato et al., 2012). The bios of these profiles typically include fictional descriptions of the character, often referencing religion (e.g., Koon & Yoong, 2017), hobbies (Kopp et al., 2015), desired partner traits (Pizzato et al., 2012), or the (fictional) reason for seeking a relationship (Anesa, 2020).

#### Scene 2. Target Selection (the “Hunt”)

After the preparation, scammers proceed to target selection. This can be *targeted* (*2a*), where individuals are chosen based on specific characteristics such as wealth, Western nationality (Suarez-Tangil et al., 2019), or signs of vulnerability such as being divorced or widowed (Khader & Yun, 2017). Alternatively, scammers may use the “*scattergun approach*” (*2b*), contacting large numbers of users through mass messages or emails (Rege, 2009, 2013), or by liking a large number of profiles in a short period on dating apps (Choi et al., 2024). In addition, some scammers use “*sucker lists*” (*2c*)—lists of past victims’ contact details, often sold between scammers (Whitty, 2013, 2023). Finally, some scammers engage in *revictimization* (*2d*), targeting their own previous victims again (Anesa, 2020; Whitty, 2015).

#### Scene 3. Initial Contact (the Hook)

Once a target is identified, the scammer initiates contact (e.g., Aborisade et al., 2024; Rege, 2009)—although in some cases, the target makes the first move (Wang & Dickinson, 2024). Initial contact occurs via various channels: *online dating apps* (*3a*) such as Tinder, eHarmony and OKCupid (e.g., Andoh-Baidoo et al., 2024; Archer, 2017; Cross et al., 2018; Kamaruddin et al., 2020); *social media platforms* (*3b*) such as Facebook, Instagram and LinkedIn (Aborisade et al, 2024; Anesa, 2020; Cross & Holt, 2021); *email* (*3c*) (e.g., Kamaruddin et al., 2020)—where scammers sometimes purchase lists of email addresses (linked to data theft; Rege, 2009); *websites and forums* (*3d*) such as Craigslist or forums for widowed individuals (Archer, 2017; Huhn, 2023); and even through *online games* (*3e*; Andoh-Baidoo et al., 2024).

##### Cross-Cutting Action for Scenes 2 and 3: Reconnaissance

In parallel with scenes 2 and 3, scammers may carry out *reconnaissance*, gathering as much information as possible about their targets. This includes examining dating profiles, social media accounts, and other online sources. The collected information is then used to tailor the scammer’s profile and contact strategy to the specific characteristics of the target (Dickerson et al., 2020; Wang & Zhou, 2023).

#### Scene 4. Transition to Private Channel (the Shift)

When contact begins on public platforms such as dating apps or social media, scammers almost always try to shift the conversation to private channels. This includes *instant messaging apps* (*4a*) like Whatsapp or Google Chat (e.g., Cross & Lee, 2022; Niman et al., 2023; Smeitink, 2021; Whitty, 2023) or *email* (*4b*) (e.g., Dickinson & Wang, 2024; Gillespie, 2017). This move helps isolate the target and avoid the monitoring and reporting features of public platforms, making it more difficult to identify and report the scam (Cross et al., 2018).

#### Scene 5. Grooming

The grooming scene plays a crucial role in building trust and emotional commitment between the scammer and the target. The goal is not only to create a strong emotional bond but also to isolate the target, making them more susceptible to future financial requests. This scene involves a wide range of actions, often carried out simultaneously or in sequence.

One key action is the *expansion of the backstory* (*5a*), a continuation of the narrative framework established earlier in 1.4b (this is indicated in the crime script with a feedback arrow). This backstory often serves as the foundation for future financial requests, such as references to family or medical situations (Choi et al., 2024; Coluccia et al., 2020; Huhn, 2023). Scammers may also introduce third parties, such as “family members” or a “lawyer,” who sometimes even engage directly with the victim (Koon & Yoong, 2017). Another typical grooming technique is the *emphasis on similarities with the victim* (*5b*), in which scammers adapt their story based on the personal information shared by the victim (e.g., Anesa, 2020; Kopp et al., 2015). This information is then used to further develop and personalize the scammer’s constructed persona (1.4), which is reflected in the crime script with another feedback arrow.

During this scene, scammers often send *gifts* (*5c*; or promise to do so) to build trust (Niman et al., 2023; Shaari et al., 2019). In some cases, these gifts are paid for using stolen credit card data from previous victims (Rege, 2009). Scammers may also introduce *(video)calling* (*5d*) as a means of communication (e.g., Abubakari, 2024; Cassiman, 2019; Newell, 2021), sometimes employing deceptive techniques such as “ID spoofing” or voice modulation software to conceal their identity (Smeitink, 2021; Cassiman, 2019).

Other actions in this scene include: *Sending documents and photos* (*5e*) to enhance credibility and connection (Dickerson et al., 2020; Smeitink, 2021); establishing regular *communication routines* (*5f*) to maintain frequent contact with the victim (e.g., Aborisade et al., 2024; Cross et al., 2018; Whitty, 2013); *expressing romantic feelings* and visions of a shared future (*5g*; Coluccia et al., 2020; Huhn, 2023)—sometimes even proposing marriage (Archer, 2017; Whitty, 2023); planning *physical meetings* (*5h*; Aborisade et al, 2024; Gillespie, 2017); or exchanging intimate photos and messages (i.e., *sexting, 5i*; Abubakari, 2024; Amirkhani et al., 2024). In some cases, these materials are saved with the intention of using them later for *sextortion* (see 8a). This is shown in the crime script with a feedforward arrow.

Another technique used to build trust and emotional dependence during the grooming phase is “*love bombing*” (*5j*), in which the scammer overwhelms the victim with intense, repeated expressions of affection in a short period of time (Koon & Yoong, 2017). This can emotionally disarm the victim and reduce the time and space needed for rational financial decision-making (Cross et al., 2018). Scammers also apply *isolation techniques* (*5k*). For example, they may ask the victim to keep the relationship secret from friends and family (Cross et al., 2018; Whitty, 2023), often under the pretense of protecting the relationship from outside judgment. They may also display *controlling or possessive behavior* (*5l*) to further dominate the victim, demanding constant availability or asking the victim to justify how they spend their time when not communicating with the scammer (Aborisade et al., 2024).

#### Scene 6. The Sting

When the scammer senses that enough trust and emotional commitment has been established, they transition into what is often referred to as the “*sting*”: the scene in which the victim is financially exploited. This exploitation can take various forms, with three main strategies that are often used individually, in combination, or in sequence to *request money* (*6.1*).

A common approach is to begin by requesting *small amounts of money* (*6.1a*). This is often used to test the victim’s willingness and to lower the threshold for giving larger sums—a technique known as the “*foot-in-the-door*” technique (Whitty, 2013). These small requests may relate to personal items like a laptop or phone (Whitty, 2015), travel expenses such as a passport or airline ticket (Coluccia et al., 2020; Kamaruddin et al., 2020), or upfront fees allegedly needed to unlock a larger payout, such as an inheritance (Cross & Holt, 2021; Wang & Topalli, 2024b).

When *larger amounts* are requested (*6.1b*), they often involve high costs tied to complex and escalating scenarios. These may include medical expenses (Anesa, 2020; Dickinson & Wang, 2024), financial support needs (Choi et al., 2024; Smeitink, 2021), or investment opportunities where the victim is convinced to invest in a fictitious company—a tactic related to 419 scams and advance fee fraud (Lazarus et al., 2023; Smeitink, 2021).

In many cases, scammers employ a *crisis narrative* (*6.1c*) to justify both small (6.1a) and large (6.1b) requests, and to lay the groundwork for future financial demands. These narratives focus on urgent, fabricated situations where immediate financial support appears absolutely necessary. For instance, medical expenses may be attributed to a serious car accident, and travel costs may be linked to made-up customs issues. What distinguishes the crisis narrative is its intense emphasis on urgency, which significantly increases pressure on the victim.

As in the grooming scene, scammers may involve *third parties* (*6.2*) during the exploitation scene to reinforce credibility and urgency. These figures may pose as authority figures—such as doctors (after a supposed accident), lawyers, or customs officials. Sometimes, fictional “family members” of the scammer are also introduced to further persuade the victim (Archer, 2017; Cross & Holt, 2021; Cross & Lee, 2022; Wang & Zhou, 2023).

##### Cross-Cutting Actions for Scene 6: Escalation and Promises

In many cases, the excuses scammers use to ask for money escalate over time, both in severity and financial demand (Whitty, 2015). Victims are almost always promised that they will get their money back (Koon & Yoong, 2017; Wang & Zhou, 2023), or in the case of advance fee or investment scams, that they will even share in the scammer’s supposed profits (Wang & Zhou, 2023). These promises help reinforce the victim’s trust, leading many to continue sending money despite earlier doubts or setbacks.

#### Scene 7. Financial Transaction (the Payout)

Once the victim is convinced to financially support the scammer, the transaction can take place in several ways. *Payments* (*7.1*) may be made via *bank transfers* (*7.1a*; Huhn, 2023); *money transfers* (*7.1b*) through money transfer operators (MTO) such as Western Union or MoneyGram (Abubakari, 2024; Cross & Lee, 2022; Smeitink, 2021; Whitty, 2023); *gift cards* (*7.1c*) for platforms such as Amazon or iTunes (Cross & Lee, 2022; Dickinson et al, 2023; Huhn, 2023); *cryptocurrency* (*7.1d*; Cross, 2023; Wiederhold, 2024); or through a *fake app* developed by scammers (*7.1e*), such as “Huobi” (Wang & Zhou, 2023).

These payments may also lead to *money laundering practices* (*7.2*) intended to conceal the criminal origin of the funds. In some cases, the victim is actively involved in the laundering process, for example, by forwarding money to other accounts on behalf of the scammer (Andoh-Baidoo et al., 2024; Huhn, 2023), or by emptying their own bank accounts and converting the funds into cash or property (Huhn, 2023). In such scenarios, the victim functions as a money mule, and in some cases may even be considered a co-offender (Huhn, 2023).

##### Cross-Cutting Action for Scenes 6 and 7: Returning to Grooming in Case of Resistance

There are instances in which the victim begins to question the scammer’s financial requests or fabricated stories. In such cases, scammers often revert to previously used grooming strategies to rebuild trust and increase the victim’s compliance (Whitty, 2013). This dynamic is represented in the crime script with a feedback arrow. While the exact tactics used in these situations are not extensively detailed in the reviewed literature, it is likely that specific techniques—depending on the nature of the conflict or hesitation—are emphasized more strongly.

Additionally, scammers employ various forms of emotional manipulation to reinforce obedience and suppress resistance, including: Inducing guilt (e.g., *“If you really loved me, you’d do this for me”*; Huhn, 2023); threatening to end the relationship (Archer, 2017); threatening self-harm (Wang & Topalli, 2024b); using *silent treatment*, that is, abruptly stopping communication to make the victim feel anxious and insecure (Cross et al., 2018); and, in some cases, threatening physical violence (Archer, 2017).

#### Scene 8. The Squeeze

At a certain point, the victim may refuse to make further payments or may no longer have the financial means to support the scammer. In such cases, scammers may shift to new strategies to continue exploiting their victim. This scene is referred to as “the squeeze,” as the scammer attempts to extract the last bit of value before the scam comes to an end.

One of these strategies is *sextortion* (*8a*), where the scammer coerces the victim into sending sexually explicit images or uses previously shared intimate material to blackmail them (Anesa, 2020; Amirkhani et al., 2024; Cross, 2023; Cross et al., 2024; Cross & Lee, 2022; Whitty, 2015). Another method is *non-sexual blackmail or extortion* (*8b*), for example, when the victim has been drawn into money laundering schemes (Cross, 2023) or even drug smuggling (Whitty, 2023). Finally, the scammer may *threaten the victim or their loved ones* to force additional payments (*8c*; Choi et al., 2024; Shaari et al., 2019).

#### Scene 9. The Aftermath

Eventually, the victim appears to stop making payments for good. Even at this point, scammers may still deploy various strategies to achieve *revictimization* (*9.1*). One such strategy involves the scammer *admitting the scam*, but claiming to have developed genuine feelings for the victim, thereby creating the illusion of a *real relationship* (*9.1a*; Anesa, 2020; Whitty, 2015). In this supposedly authentic relationship, the scammer often resumes financial requests.

Another common tactic is the *recovery scam* (*9.1b*), in which the scammer poses as a third party offering to help the victim recover their lost money (Smeitink, 2021; Whitty, 2015). In both cases (9.1a and 9.1b), the scammer targets the victim once more, creating a feedback loop that leads back to the target selection scene (action 2d) to continue or restart the scam. This loop is represented in the crime script with two feedback arrows.

A third revictimization strategy is the sharing of victim information with other scammers via the aforementioned *sucker lists* (*9.1c*; Whitty, 2013, 2023). Unlike the previous strategies, this form of revictimization does not create a feedback loop in the script, as the original scammer adds the victim to a sucker list for use by others—without intending to initiate contact again themselves.

However, revictimization is not always successful (or attempted). At that point, the scam reaches its *definitive end* (*9.2*). In some cases, the scammer stops all contact without explanation, a tactic often described as *ghosting* (see Schokkenbroek et al., 2025; *9.2a*). In other instances, the scammer is *exposed*, and the victim ends the relationship themselves (*9.2b*).

## Discussion

This study provides a comprehensive and systematic overview of the complex methods behind online romance scams, captured in a detailed crime script. By breaking down the entire criminal process into distinct scenes and actions, the script reveals how scammers methodically build trust, create emotional dependency, and ultimately exploit their victims financially. The crime script also highlights how scammers target individual vulnerabilities, contributing to the destructive emotional and financial impact of this type of fraud. Table 2 provides a summary of the critical findings of this study.

**Table 2.** Summary of Critical Findings.

Critical Findings
• **Online romance scams follow recurring yet flexible patterns:** Scammers use a dynamic, non-linear process to manipulate victims, often revisiting previous steps (e.g., grooming) to regain trust.
• **Grooming involves systematic emotional manipulation:** Scammers employ love bombing, isolation, fabricated crises, and deep personalization to create dependency and weaken resistance.
• **Financial exploitation is varied and escalating:** This includes small and large requests, urgent crises, involvement of fake third parties, and repeated transactions.
• **Scammers may use advanced technologies and deceptive strategies:** This includes deepfakes, spoofed calls, AI-generated content, and identity theft to build credibility and evade detection.
• **Revictimization is a common risk:** Victims are often re-targeted through recovery scams, sucker lists, or re-initiated contact by scammers under new identities.
• **Crime scripting is an effective tool to map modus operandi:** It offers a comprehensive and adaptable framework to understand scam strategies and inform prevention.

One of the most striking findings is the dynamic and flexible nature of these scams. Scammers are highly adaptive, continuously adjusting their approach to fit the personality, behaviors, and needs of their victims. This adaptability is also evident in the iterative strategies they employ. For example, when a victim resists financial requests, scammers often revert to earlier stages, such as grooming, to rebuild trust. This cyclical process sets online romance scams apart from most other forms of fraud and underscores the complexity of these deceptive tactics.

The study also sheds light on the varied forms of revictimization that victims may experience. Victims are not only re-targeted by the same scammers but also by others, for example through sucker lists, where their contact information is sold to other (potential) offenders. Additionally, victims often become targets of follow-up fraud, including blackmail, sextortion, or recovery scams—in which criminals pose as third parties who promise to recover lost funds but in fact further exploit the victim. These forms of further victimization reveal how scammers exploit victims’ ongoing vulnerability, even after the original scam has ended.

While our crime script confirms previous findings that online romance scams often follow certain core scenes—such as initiation, grooming, and financial exploitation (e.g., Huhn, 2023; Shaari et al., 2019; Whitty, 2015)—it also demonstrates that these scams rarely follow a fixed, linear pattern. Instead, the script emphasizes the non-linear and iterative structure of this type of fraud, in which scenes may be repeated and actions adjusted based on the victim’s response. This illustrates how difficult these scams are to detect and disrupt, while also showing that multiple intervention points exist throughout the process.

With these insights, our crime script offers both a valuable theoretical contribution to the understanding of online romance scams and concrete, practical guidance for policy and practice, such as identifying opportunities for better detection and shaping effective prevention campaigns. As such, the script provides a solid foundation for the further development of (preventive) interventions and policy aimed at better protecting victims and disrupting these fraudulent schemes.

### Limitations

While this study provides valuable insights into the process behind online romance scams, there are several limitations that should be acknowledged. First, our model is based solely on findings from existing research, making it dependent on the quality and scope of those sources. As a result, certain scammer strategies may have been overlooked if they fell outside the scope or findings of the included studies. This risk is amplified by the fact that most of the examined studies did not obtain their data directly from offenders. The information in these studies is primarily drawn from victims, experts (such as investigators), legal case documentation, or—in very few instances—online ethnographic research or interviews among perpetrators. This inevitably shapes the granularity and perspective of the findings. While such sources offer valuable insights into scammers’ behaviors as experienced by others, they may not fully capture how scammers themselves make decisions, adapt strategies, or experience constraints during the scam process. This is particularly relevant for understanding specific tracks within the crime script—that is, the actual combinations and sequences of actions chosen by offenders in practice—which would benefit from direct insights into offender reasoning and operational logic. Future research could address this gap by exploring ethically sound and methodologically rigorous ways of accessing scammer perspectives. Possible approaches include online ethnography in scammer forums or encrypted messaging spaces, language analysis of scammer communications, or collaborations with law enforcement to analyze interrogation records or case files. These methods could provide a more detailed view of how scammers navigate different decision points and how contextual, organizational, or psychological factors shape their tracks through the crime script. Furthermore, to fully understand the business models behind these modus operandi as well as the interconnectedness with financial-economic forms of crime (such as specific forms of money laundering), it is recommended to use financial crime scripting (Snaphaan & Van Ruitenburg, 2025) in future studies.

Second, the findings of our scoping review are only as diverse and inclusive as the studies included. A notable limitation of the reviewed literature is the demographic and geographic skew in available research. As shown in Appendix I, the majority of included studies originate from the United States, Australia, and the United Kingdom, with limited empirical attention to (and from) other regions, cultural settings, specific (online) subcultures, or marginalized populations. As a result, the experiences of, for example, LGBTQIA+ individuals, racial and ethnic minorities, older adults, and users of non-mainstream platforms remain largely underexplored. For example, none of the included studies investigated experiences of LGBTQIA+ victims or the strategies specifically targeting them or online platforms tailored to them, such as LGBTQIA+-oriented dating apps. This is notable given that media reports indicate heightened vulnerability among LGBTQIA+ individuals to online romance scams, often in combination with sextortion (Kreidler, 2022; Skiba, 2021). This limited empirical attention towards other regions, cultural settings, or populations limits our understanding of how scammers may tailor their tactics—for instance, by crafting culturally specific personas or targeting groups with limited access to support services. Some studies in our review suggest that scammers’ backstories and emotional appeals are adapted to local contexts or victim characteristics, but the evidence is still sparse. Addressing this gap is crucial not only for creating more inclusive and representative crime scripts, but also for developing targeted prevention strategies and victim support services that account for diverse vulnerabilities and platform ecologies. Future research should address this gap by incorporating more inclusive and intersectional approaches to better reflect the full spectrum of experiences with online romance scams.

Third, another limitation is that the crime script we developed has not yet been empirically validated. Further research is necessary to test the script in various contexts—for example, by comparing it to court documents, conducting interviews or surveys with offenders (where possible), victims, experts, support organizations, or law enforcement agencies. Additionally, future (experimental) research should investigate to what extent the script can be used to develop effective (preventive) intervention strategies.

Finally, we must also consider the shelf life of the crime script. Online romance scams continue to evolve, with scammers increasingly adopting new technologies and tactics to deceive victims. For instance, recent cases have revealed that some scammers now use (generative) artificial intelligence in the initial contact phase (see, e.g., Goodwin, 2024), including deepfake-powered video calls and automated chatbots. Despite this, empirical evidence on scammers’ use of more advanced technologies is still limited. However, given the rapid pace of technological change and the history of scammer adaptability, such developments are worth anticipating. For example, voice AI could allow scammers to simulate real-time phone conversations with greater emotional believability, and the potential expansion of metaverse or VR spaces may eventually offer new environments for romantic deception. Similarly, if scammers were to exploit dating app APIs or automation tools, they could scale their targeting efforts more efficiently and evade platform safeguards. While these developments remain largely speculative at this stage, they underscore the need for continued monitoring of scammer strategies. Thus, while our crime script already incorporates some technological innovations, ongoing revisions will be essential to ensure the model remains responsive to emerging scam tactics and evolving digital environments and possibilities.

### Scientific and Societal Implications

This study contributes to our understanding of online romance scams and digital fraud more broadly by applying crime script analysis as a framework. Our findings demonstrate the usefulness of crime scripting in mapping out complex, digital, and interpersonal forms of crime and also emphasize how dynamic and iterative criminal behavior can be. While previous studies have applied crime scripting to various types of cybercrime (see Dehghanniri & Borrion, 2021), such as phishing (Loggen & Leukfeldt, 2022) or customer-to-customer e-commerce fraud (Lee, 2022), none of them focused on online romance scams. As a relatively large number of studies has provided insights into (aspects of) the modus operandi of online romance scammers, a step-by-step synthesis of scammers’ behaviors and strategies was possible—and warranted. By applying crime script analysis to this existing body of knowledge, we consolidate available insights and map the scam process as a whole. We argue that this crime script is more than the sum of its parts; it generates unique insights that provide a rich foundation for further theoretical exploration of scammers’ strategies. Our study demonstrates that crime scripting can be a useful analytical method when combined with a scoping literature review to synthesize existing knowledge on specific forms of crime.

In addition to theoretical insights, the crime script provides practical tools for interventions and policymaking (for an overview, see Table 3). By breaking the scam process down into individual scenes and actions, policymakers and practitioners can develop targeted measures that respond to specific elements of a scam. For example, our findings highlight the frequent use of payment methods such as Western Union and gift cards for platforms like Amazon and iTunes (Huhn, 2023; Whitty, 2023). These insights can inform targeted prevention campaigns, such as displaying warnings at the point of purchase or during transactions, and training retail employees to recognize red flags.

**Table 3.** Summary of Implications for Practice, Policy, and Research.

	Implications
**Practice**	• **Use detailed scam patterns in awareness and prevention tools:** Prevention campaigns should reflect the full scam trajectory, including the transition to private channels, emotional manipulation techniques, revictimization tactics, and the squeeze scene (e.g., sextortion). • **Informed decision-making regarding interventions and its design:** The crime script offers a structured understanding of the criminal process, enabling security actors to identify where the script intersects with their operational responsibilities, and how they can intervene effectively.
**Policy**	• **Encourage collaboration with digital platforms and financial institutions:** Social media, dating platforms, banks, and money transfer services should collaborate on early detection strategies (e.g., recognizing scam profiles, suspicious payment activity) and user alerts.
	• **Balance fraud prevention with online inclusivity and privacy:** While digital identity verification is crucial to fraud reduction, efforts should account for users who require anonymity, such as individuals exploring gender identity or in vulnerable contexts.
**Research**	• **Empirically validate the crime script across diverse contexts:** Further research is needed to test the crime script with real-world data (e.g., court cases, law enforcement interviews) to assess its generalizability and practical value.
	• **Track evolving scam strategies, especially technological innovations:** Scams are rapidly changing, with increasing use of AI, deepfakes, and automation. The crime script should be treated as a living tool, regularly updated to reflect these developments.

Moreover, training programs and detection technologies could be developed to identify specific elements of scammers’ methods, such as the use of deepfakes or manipulative grooming techniques. By better equipping victims and professionals with knowledge about these patterns, interventions can be more effective. Our findings also show that scammers almost always use fake profiles, underlining the need for stronger strategies and regulations around digital identity verification. At the same time, it is essential to differentiate between profiles used for romance scams and those created without fraudulent intent.^4^ Overly strict verification requirements could pose problems for users who wish to remain anonymous for legitimate reasons, such as those exploring their gender identity or navigating sensitive social situations. Striking a balance between fraud prevention and online privacy/inclusivity is therefore crucial.

In detecting scam profiles on social media and dating apps, as well as identifying financial transactions linked to romance scams, external collaboration can be highly valuable (see also Dickerson et al., 2020). Detection systems could be enhanced through cooperation with reporting platforms such as Scamdigger.com, RomanceScams.org, and SocialCatfish.com, which collect victim testimonies and expose confirmed scam profiles. Integrating such data into the algorithms of social media and dating platforms could improve early detection of suspicious accounts and provide timely warnings to users. These platforms also play an important role in raising awareness and supporting victims in sharing their experiences and reporting scams.

Financial institutions, including banks and MTOs, can also (further) contribute to combatting online romance scams. By analyzing specific transaction patterns linked to these scams, they can flag suspicious activity early and help protect victims from further financial harm. Collaboration between financial institutions and law enforcement can further enhance the effectiveness of these strategies.

Another key recommendation from our study is the strengthening of awareness and resilience among social media and dating app users. By mapping the modus operandi of romance scammers in detail, the crime script offers valuable insights that can be translated into practical tools. As previously suggested by Khader and Yun (2017), a checklist of recognizable scammer strategies could be developed and integrated into the account registration process of social media and dating apps. This would raise awareness of common scams from the outset and help users protect themselves (Eseadi et al., 2021). Such measures not only serve a preventive function but also enhance users’ overall digital literacy.

Last, considering the transnational nature of online romance scams—where offenders often operate from countries like Nigeria, Ghana, and Malaysia, while victims are predominantly located in Western countries (Suarez-Tangil et al., 2019)—an international approach is essential. This includes not only the exchange of information between countries but also joint efforts in detection, investigation, prosecution, and prevention. International cooperation can help deliver a more coordinated and effective response to this complex and evolving form of fraud.

## Conclusion

This study offers a detailed understanding of the methods used by online romance scammers through the lens of crime script analysis. The developed script reveals how scammers strategically and flexibly exploit emotional vulnerabilities and technological opportunities to manipulate and financially exploit their victims. By breaking down the scam process into scenes and (equifinal) actions, this model provides not only theoretical insights but also concrete entry points for policy and practice. At the same time, it highlights the need for further research, such as empirical validation and investigations into (technological) innovations in scammers’ methods—ideally studied from the perspective of offenders themselves.

Online romance scams exemplify how vulnerabilities in both online systems and human emotions and desires can be exploited. Our crime script helps to map these vulnerabilities—and in doing so, offers a crucial first step in untangling these heartbreaking schemes.

## Supplemental Material

sj-docx-1-tva-10.1177_15248380251361046 – Supplemental material for Love as Bait: A Scoping Review and Crime Script Analysis of Online Romance Scams Supplemental material, sj-docx-1-tva-10.1177_15248380251361046 for Love as Bait: A Scoping Review and Crime Script Analysis of Online Romance Scams by Janneke M. Schokkenbroek and Thom Snaphaan in Trauma, Violence, & Abuse

## Notes

1. Although romance scam models typically refer to “phases,” crime script analysis uses the term “scenes” (see section “Crime Scripting”). For the sake of consistency, we will henceforth use the term “scenes.”

2. Please note that in the crime script analysis, we use the term “target” in the early scenes—from preparation through the initiation of contact—as this term reflects that the individual has been selected as the aim of a deceptive effort but has not yet necessarily experienced harm. Beginning with the grooming scene, we transition to the term “victim,” as this marks the stage at which psychological manipulation and emotional harm can already begin to occur, even before financial exploitation. The term “user” is used only in platform-specific contexts (e.g., “dating app user”), where it refers to general platform activity.

3. For an example of such a scammer script or “playbook,” see SocialCatfish (2023).

4. See Schokkenbroek (2024) for an overview of non-financial motivations behind the use of fake profiles and catfishing.
