# Supplementary material for: Love as Bait: A Scoping Review and Crime Script Analysis of Online Romance Scams
Source: Trauma Violence Abuse. 2025 Sep 9;27(4):1877–93. doi: 10.1177/15248380251361046 (PMC13578658; doi:10.1177/15248380251361046)
Supplement: Supplementary file 1 [file sj-docx-1-tva-10.1177_15248380251361046.docx]

**Supplementary Material for the Manuscript (R1) entitled ‘Love as Bait: A Scoping Review and Crime Script Analysis of Online Romance Scams’**

**Appendix I. Summary of Included Records**

| **Author(s) & Year of Publication** | **Country of Researcher(s)’ Affiliation** | **Geographical Scope of the Study ^†^** | **Year (included in) Data Collection** | **Research Method + Data source(s)** | **Sample** | **Type of Publication** |
| --- | --- | --- | --- | --- | --- | --- |
| Aborisade, Ocheja, & Okuneye, 2024 | Nigeria | Victims from six countries (Canada, Germany, Ireland, Sweden, United Kingdom, United States), victimized by Nigerian scammers | 2023 | Semi-structured video interviews with victims from online romance scams | 10 victims of online romance scams were interviewed | Journal article |
| Abubakari, 2024 | Poland | Ghana | Not specified | Interviews with female perpetrators of online romance scams | 15 female romance scam perpetrators | Journal article |
| Amirkhani, Alizadeh, Randall, & Stevens, 2024 | Germany | Iran | Not specified | Narrative interviews with victims of online dating fraud with sexual scam experience | 20 victims of online dating fraud | Conference Proceedings |
| Andoh-Baidoo, Offei, Ayaburi, Siponen, & Gladyshev, 2024 | United States; Ghana | Not specified (Africa) | Not specified | Document analysis of published articles on online romance scams and grey literature; unspecified | Not specified | Conference Proceedings |
| Anesa, 2020 | Italy | Malaysia; South Africa | 2010-2018 | Two data sources: 1) Text messages between scammer and victim obtained via Malaysian authorities; 2) scam template messages related to the U.S. case Olayinka Ilumsa Sunmola. | 26 messages from scammer to victim + 43 scam templates | Journal article |
| Archer, 2017 | United States | Not specified | 2014-2016 | Analysis of romance scam victim testimonials collected from a public, online blog | 82 victim testimonials | Thesis |
| Barnor, 2024 | Ghana | Ghana | Not specified | Semi-structured interviews with cybercrime perpetrators and law enforcement officials | 8 perpetrators from 2 Ghanian cybercrime hotbeds + 3 law enforcement officials | Journal article |
| Barnor, Boateng, Kolog, & Afful-Dadzie, 2020 | Ghana | Ghana | Not specified | Semi-structured interviews with romance scam perpetrators | 10 romance scam perpetrators were interviewed | Conference Proceedings |
| Cassiman, 2019 | Belgium | Ghana | 2014-2016 | Fieldwork in Ghana consisting of conversations and interviews | Not specified | Journal article |
| Choi, Lee, & Choi, 2024 | South Korea | South Korea | 2023 | 1) Document analysis of publicly accessible online data via South Korean search engine and YouTube; 2) Expert interviews with investigators via e-mail | 1) Not specified (437 KB file volume); 2) 5 investigators with expertise in probing romance scams were interviewed | Journal article |
| Coluccia, Pozza, Ferretti, Carabellese, Masti, & Gualtieri, 2020 | Italy | Not specified | Until December 2019 | Document analysis of published articles on online romance scams | 12 records included | Journal article |
| Cross, 2022 | Australia | Not specified | Not specified | Document analysis of published articles and law enforcement websites to analyze the role of AI in romance scams | Not specified | Journal article |
| Cross, 2023 | Australia | Australia | 2018-2019 | Analysis of victims' online romance scam reports, obtained from the Australian Consumer and Competition Commission (ACCC) | 1015 victim testimonials that included reports on triggers to a realization of romance fraud victimization | Journal article |
| Cross & Holt, 2021 | Australia | Australia | 2018-2019 | Analysis of victims' online romance scam reports, obtained from the Australian Consumer and Competition Commission (ACCC) | 375 victim testimonials that included reports on military fraud | Journal article |
| Cross & Holt, 2023 | Australia | Australia | 2018-2019 | Analysis of victims' online romance scam reports, obtained from the Australian Consumer and Competition Commission (ACCC) | 2699 victim testimonials | Journal article |
| Cross & Lee, 2022 | Australia | Australia | 2018-2019 | Analysis of victims' online romance scam reports, obtained from the Australian Consumer and Competition Commission (ACCC) | 104 victim testimonials that included reports on fear of crime | Journal article |
| Cross, Dragiewicz, & Richards, 2018 | Australia | Australia | Not specified | Semi-structured interviews with victims of online romance scam(s) | 21 romance scam victims were interviewed | Journal article |
| Cross, Holt, & Holt, 2023 | Australia; United States | Australia | 2018-2019 | Analysis of victims' online romance scam reports, obtained from the Australian Consumer and Competition Commission (ACCC) | 210 victim testimonials that included reports on sextortion | Journal article |
| Cross, Holt, & O'Malley, 2024 | Australia; United States | Australia | 2018-2019 | Analysis of victims' online romance scam reports, obtained from the Australian Consumer and Competition Commission (ACCC) | 258 victim testimonials that included reports on sextortion | Journal article |
| Dickerson, Apeh, & Ollis, 2020 | United Kingdom | Not specified | Not specified | Experimental study, a mock dating platform prototype was developed to test romance fraud detection and awareness | 200 respondents | Conference Proceedings |
| Dickinson & Wang, 2024 | United States | Not specified | 2022 | Experimental study and linguistic analysis: listed scammers were emailed and their responses were analyzed | 87 online romance scammer e-mail exchanges | Journal article |
| Dickinson, Wang, & Maimon, 2023 | United States | Not specified | 2020-2021 | Experimental study and linguistic analysis: listed scammers were emailed and their responses were analyzed | 94 scammer responses | Journal article |
| Edwards, Peersman, Stringhini, Rashid, & Whitty, 2018 | United Kingdom | Global | Until March 2017 | Analysis of romance scammer profile data obtained from a public online dating scamlist maintained at scamdigger.com | 5402 scam profile instances | Conference Proceedings |
| Gillespie, 2017 | United Kingdom | Not specified | Not specified | Document analysis of published articles on online romance scam and of court cases | Not specified | Journal article |
| Huang, Stringhini, & Yong, 2015 | Australia; United Kingdom; China | China | 2012-2013 | Analysis of scam accounts detected by a dating site +(anonymized) | > 500,000 scammer accounts | Conference Proceedings |
| Huhn, 2023 | United States | United States | Various specified | Two data sources: 1) Field interviews with victims of online romance scams + money mule practices; 2) Analysis of three closed criminal investigations | 1) Three victims were interviewed; 2) Three closed criminal investigation cases were analyzed | Thesis |
| Kamaruddin, Wan Rosli, Abd Rani, Md Zaki, & Omar, 2020 | Malaysia | Not specified | Not specified | Document analysis of legal and regulatory frameworks on online romance scams | Not specified | Journal article |
| Khader & Yun, 2017 | Singapore | Not specified | Not specified | Document analysis of published articles on online romance scams and grey literature (e.g., law enforcement websites, popular media) | Not specified | Book chapter |
| Kopp, Layton, Sillitoe, & Gondal, 2015 | Australia | Europe | Not specified | Analysis of fraudulent profiles published on a dating site as a list of known scam profiles | 37 fraudulent profiles from dating site | Journal article |
| Newell, 2021 | Belgium | Ivory Coast | 2015 | Ethnographic study of online romance scammers in Ivory Coast | Not specified | Journal article |
| Niman, Parulian, & Rothhaar, 2023 | Indonesia; United States | Indonesia | Not specified | Semi-structured interviews with Indonesian female victims of online romance scams | 12 female Indonesian victims of romance scams were interviewed | Journal article |
| Pizzato, Akehurst, Silvestrini, Yacef, Koprinska, & Kay, 2012 | Australia | Australia | Not specified | Historical data; Profile information from a major Australian online dating website; Expression of Interest messages from profiles were analyzed for suspicious activity | > 2 million messages | Conference Proceedings |
| Rege, 2009 | United States | Global | 2000 - 2009 | Document analysis on data on online romance scams from dating sites, news and media sites, anti-scam commissions, law enforcement agencies and government agencies; retrieved from Google | 170 documents | Journal article |
| Rege, 2013 | United States | Not specified | 2000 - 2013 | Document analysis on news sites, anti-scam sites, computer and security sites, government documents, online dating review/magazine sites, academic journals, and books; collected through Google Search, Google Scholar, and Temple University libraries. | 18 case studies | Conference Proceedings |
| Shaari, Kamaluddin, Fauzi, & Mohd, 2019 | Malaysia | Malaysia | Not specified | Linguistic analysis of online communication between scammers and victims, obtained via a Facebook page (' Romance scam research Malaysia') and through collaboration with the Malaysian Police Department | 30 sets of online communication | Journal article |
| Smeitink, 2021 | The Netherlands | The Netherlands | 2020 | Various research methods: 1) Interview study with victims and experts of online romance scams; 2) Online observations of Facebook pages; 3) Online experiment with fake profile to lure online romance scammers and test their tactics | 1) Nine victims and four experts of online romance scams were interviewed; 2) Not specified; 3) Not specified | Thesis |
| Suarez-Tangil, Edwards, Peersman, Stringhini, Rashid, & Whitty, 2019 | United Kingdom; Australia | Global | Until March 2017 | Analysis of romance scammer profile data obtained from a public online dating scamlist maintained at scamdigger.com | 14720 ordinary profiles vs 5402 scammer profiles | Conference Proceedings |
| Tan, & David, 2013 | Malaysia | Malaysia | Not specified | Case study of an online romance scam, data (e-mails) obtained from the RMP’s Cybercrime and Multimedia Investigation Unit as part of a closed online romance scam case | 21 e-mails (15 e-mails from scammer, 6 e-mails from victim) | Journal article |
| Wang, 2024 | United States | China (data source); Myanmar (location of crime syndicates) | Not specified | Analysis of victim testimonials from online Chinese platforms (Zhihu and WeChat) | 36 victim testimonials | Journal article |
| Wang & Dickinson, 2024 | United States | Not specified | 2020-2021 | Experimental study and linguistic analysis: listed scammers were emailed and their responses were analyzed | 94 scammer responses | Journal article |
| Wang & Topalli, 2024a | United States | United States | Not specified | Document analysis of open source data (via Google Search) from online platforms that gathered employees' reviews, thoughts, or experiences regarding chat moderator companies | 69 review posts (38 Glassdoor; 16 Trustpilot; 15 Reddit) | Journal article |
| Wang & Topalli, 2024b | United States | Global | Not specified | Analysis of victim testimonials of online romance scam experiences, collected from multiple web-based sources | 52 victim recounts | Journal article |
| Wang & Zhou, 2023 | United States; China | China | 2021 | 1) Victims’ testimonial data collected from Zhihu, a prominent Chinese online platform for posting questions and answers and users’ personal experiences; 2) Analysis of complete police reports from law enforcement in China | 1) Testimonials of 40 victims; 2) 4 police reports | Journal article |
| Wang & Lu, 2007 | United States | China | 2003 | Case study of an online romance scam, originated from a major Chinese website | 1 case study | Journal article |
| Whitty, 2013 | United Kingdom | United Kingdom | Not specified | Semi-structured interviews with victims of online romance scam(s) | 20 romance scam victims were interviewed | Journal article |
| Whitty, 2015 | United Kingdom | United Kingdom | Not specified | Study 1: posts from public website in which victims (financial and non-financial) stated their online romance scam experiences; Study 2: in-depth interviews with victims of online romance scams; Study 3: interview with SOCA (Serious Organised Crime Agency) officer who was an expert in law enforcement regarding online romance scams | Study 1: 200 posts; Study 2: 20 victims were interviewed; Study 3: 1 SOCA officer was interviewed | Journal article |
| Whitty, 2019 | Australia; United Kingdom | United Kingdom | Not specified | Online survey examining online romance scam detection | 261 survey respondents | Journal article |
| Whitty, 2023 | Australia | Not specified (due to anonymization of the victim) | Not specified | Case study of a victim of a romance scam who was arrested for drug trafficking | 1 victim case study | Journal article |
| Wiederhold, 2024 | United States | Not specified | Not specified | Document analysis of published articles and grey literature (news articles; government websites) | Not specified | Journal article |
| Yen & Jakobsson, 2016 | United States | United States | 2015 | Experimental study, 'honeypot' advertisements were posted on Craigslist to attract scammers; Analysis of scammer messages | 541 responses to personal ads | Book chapter |

*Note.* Records are ordered alphabetically by first author. **†** “Geographical Scope of the Study” refers to the country or countries from which the data originated or which were the focus of analysis (e.g., location of victims, offenders, or law enforcement), where specified; in many cases, however, this information is unclear or omitted due to the international and/or anonymous nature of online romance scams.

**Appendix II. Overview of Existing Process Models of Online Romance Scams**

| **Author(s) & Year of Publication** | **Model** | **Model Description** |
| --- | --- | --- |
| Whitty, 2013 | *Scammers persuasive techniques model* | Phase model illustrating the linear progression of an online romance scam, including the following stages:   (1) Motivated to find an ideal partner (2) Presented with an idealized profile (3) Grooming phase (4) Two types of manipulative techniques to request money:     a. *Foot-in-the-door technique*     b. *Crisis scenario* (5) These techniques are followed by continuation strategies:     a. *Crisis continues*     b. *No crisis*     c. *New crisis*     d. *Door-in-the-face technique* (6) Revictimization as the final stage (7) Sexual violence as a separate, peripheral element of the online romance scam |
| Whitty, 2015 | *Anatomy of the online dating romance scam* | Phase model illustrating the linear progression of an online romance scam, including the following stages:   (1) Profile creation (2) Grooming phase (3) Fraud using four different strategies:     a. *Small amounts of money → crisis* (*foot-in-the-door technique*)     b. *Crisis-based request*     c. *Crisis → reduced request* (*door-in-the-face technique*)     d. *Requests for small amounts only* (4) Sexual violence (5) Disclosure (revelation) |
| Shaari, Kamaluddin, Fauzi, & Mohd, 2019 | *Online romance scam-attack framework* | Framework describing and categorizing the various strategies used by offenders. Three main phases are distinguished, comprising a total of eight subphases. Within these phases and subphases, 16 steps are identified that categorize the scammer’s strategies:   (1) Initial Phase: Establishing contact and entering into a relationship     a. Subphase 1.1: Initiating contact     b. Subphase 1.2: Entering a relationship (2) Pre-attraction Phase: Building trust and grooming     a. Subphase 2.1: Gaining trust     b. Subphase 2.2: Developing a personal relationship     c. Subphase 2.3: Grooming (3) Hooked Phase: Setting up and executing the scam     a. Subphase 3.1: Maintaining the scam     b. Subphase 3.2: Baiting     c. Subphase 3.3: Execution |
| Whitty, 2019 | *Revised scammers persuasive techniques model* | Phase model with a linear structure. Broadly similar to the earlier version (Whitty, 2013), except that one phase has been added (i.e., detection of scam vs. authentic profile) and the persuasion strategies in the exploitation phase have been revised *(changes from Whitty (2013) are italicized)*:   (1) Motivated to find an ideal partner (2) Presented with an idealized profile (3) *Detection of scam vs. authentic profile* (4) Grooming phase (5) Two types of manipulative techniques to request money:     a. *Foot-in-the-door technique*     b. *Crisis scenario* (6) These techniques are followed by continuation strategies:     a. *Crisis continues*     b. *No crisis*     c. *New crisis*     d. *Door-in-the-face technique* (7) Revictimization as the final stage (8) Sexual violence as a separate, peripheral element of the online romance scam |
| Anesa, 2020 | *Online romance scam model* | Flowchart of the progression of an online romance scam, outlining approximately seven distinct steps:   (1) Initial contact (2) Introduction and self-description (3) Request to continue communication via instant messaging or email (4) “Dating” phase (5) Small request for money     a. If the victim *agrees*, financial requests continue until they eventually refuse     b. If the victim *refuses*, the scammer may attempt to return to the “dating” phase or repeat the request (6) End of scam (7) Revictimization |
| Dickerson, Apeh, & Ollis, 2020 | *Attack tree of fraud process* | Decision tree of an online romance scammer. This model operates at such a high level of detail and includes so many possible branches that it is not feasible to identify a fixed number of phases. However, several key elements can be distinguished:   (1) The model begins with initial contact (the offender approaches the victim and conducts preliminary reconnaissance); (2) Communication between victim and offender (3) Deepening of the relationship (4) The offender constructs a narrative to justify financial need (5) The offender makes a financial request (6) The victim sends money |
| Huhn, 2023 | *Path model through romance scams to co-offending* | Phase model illustrating a circular and reciprocal process from victimhood to complicity, in cases where the victim becomes a money mule for the scammer. The model is presented from both the offender’s and victim’s perspective, with two main phases for each perspective, further divided into six subphases. Each step also includes various sub-elements.  **Offender Perspective**   (1) Main phase: Grooming     a. Creating a persona     b. Developing and maintaining the relationship     c. Inducing gullibility (e.g., mood manipulation, introducing third parties into the narrative) (2) Main phase: Exploitation d. Fraud e. Money laundering f. Problem-solving phase (e.g., overcoming victim resistance or obstacles to financial transfers)   **Victim Perspective**   (1) Main phase: Interaction with the persona     a. Emotional loneliness     b. Developing and maintaining the relationship     c. Evaluating credibility (2) Main phase: Assisting the persona d. Helping the perceived romantic partner e. Acting as a money mule f. Problem-solving phase (e.g., justifying actions, resolving logistical issues) |
| Wang & Zhou, 2023 | *Persuasive model Sha Zhu Pan scam* | Linear phase model specifically describing how perpetrators of the Sha Zhu Pan scam (also known as the Pig Butchering scam) persuade their victims. Sha Zhu Pan is a type of online romance scam combined with investment fraud. Although it is often carried out from China and Southeast Asia, it occurs globally and is always executed in an organized context.  The authors distinguish three main phases, comprising a total of five subphases:   (1) Hunting Stage     a. *Research*: The offender gathers information about potential victims (2) Grooming Stage (also referred to as the Nurturing Stage) b. *Reasoning*: The offender shares fabricated personal details and attempts to convince the victim of their credibility (3) Harvesting Stage (also referred to as the Sting Stage) c. *Reasoning*: Continued persuasion to build investment trust d. *Reframing*: Adjusting the narrative to sustain believability or respond to doubts e. *Real-world events*: Introducing external events to create a sense of urgency and pressure the victim into taking action |

| Andoh-Baidoo, Offei, Ayaburi, Siponen, & Gladyshev, 2024 | *Online Romance Fraud process* | Model describing the process of an online romance scam in the specific scenario where the offender(s) are part of an organized fraud group.  The model outlines five distinct phases:   (1) Client recruitment (2) Client grooming (3) Initial extortion (4) Escalated extortion (5) Threshold breakthrough |
| --- | --- | --- |
| Choi, Lee, & Choi, 2024 | *Crime script of online romance scams in South Korea* | Largely linear model describing the criminal process behind online romance scams in South Korea. Presented as a form of crime script analysis, the model distinguishes two main phases, with a total of 16 subphases (referred to as “scripts” by the authors):   (1) Pre-crime phase: Describes the preparation undertaken by criminal organizations to carry out online romance scams     a. Bringing together members of the criminal group     b. Developing dialogue structures     c. Gathering necessary resources for the scam (e.g., fake profiles, fake credentials) (2) Criminal event phase: Describes the steps scammers take during the execution of the scam, consisting of 13 subphases:     a. Showing interest in multiple individuals (potential victims)     b. Focusing on responsive individuals     c. Initiating contact with the selected target     d. Engaging in introductory conversations and small talk     e. Suggesting to move the conversation to the KakaoTalk platform     f. Asking varied questions to gather personal information about the target     g. Selecting a suitable scam scenario and steering the conversation in that direction     h. Establishing a romantic relationship     i. Making small requests that are difficult for the victim to refuse (repeated if necessary)     j. Victim agrees to the requests     k. Demanding money under the pretense of an emergency (repeated if necessary)     l. Alleviating doubts and reinforcing the victim’s trust     m. Financial loss for the victim |

*Note.* Records are ordered chronologically by publication year, with the oldest listed first.
